# Drug Resistance and the Prevention Strategies in Food Borne Bacteria: An Update Review

**DOI:** 10.15171/apb.2019.041

**Published:** 2019-08-01

**Authors:** Fataneh Hashempour-Baltork, Hedayat Hosseini, Saeedeh Shojaee-Aliabadi, Mohammadali Torbati, Adel Mirza Alizadeh, Matin Alizadeh

**Affiliations:** ^1^Student Research Committee, Department of Food Science and Technology, National Nutrition and Food Technology Research Institute, Faculty of Nutrition Science and Food Technology, Shahid Beheshti University of Medical Sciences, Tehran, Iran.; ^2^Department of Food Science and Technology, National Nutrition and Food Technology Research Institute, Faculty of Nutrition Science and Food Technology, Shahid Beheshti University of Medical Sciences, Tehran, Iran.; ^3^Food Safety Research Center, Shahid Beheshti University of Medical Sciences, Tehran, Iran.; ^4^Department of Food Science and Technology, Faculty of Nutrition, Tabriz University of Medical Sciences, Tabriz, Iran.; ^5^Department of Clinical Sciences (Surgery), Faculty of Specialized Veterinary Sciences, Science and Research Branch, Islamic Azad University, Tehran, Iran.

**Keywords:** Drug resistance, Food borne pathogens, Food safety, Health, Prevention strategies

## Abstract

Antibiotic therapy is among the most important treatments against infectious diseases and has tremendously improved effects on public health. Nowadays, development in using this treatment has led us to the emergence and enhancement of drug-resistant pathogens which can result in some problems including treatment failure, increased mortality as well as treatment costs, reduced infection control efficiency, and spread of resistant pathogens from hospital to community. Therefore, many researches have tried to find new alternative approaches to control and prevent this problem. This study, has been revealed some possible and effective approaches such as using farming practice, natural antibiotics, nano-antibiotics, lactic acid bacteria, bacteriocin, cyclopeptid, bacteriophage, synthetic biology and predatory bacteria as alternatives for traditional antibiotics to prevent or reduce the emergence of drug resistant bacteria.

## Introduction

Antimicrobials have a lot of usage in medicine for human, plant and veterinary for a few decades. These substances can be used on agriculture and veterinary for different means of feed efficiency, growth improve and almost simultaneously for control, prevent and treat infections.^1,2^ Antibiotics are vital medical materials which can be natural, synthetic or semi-synthetic and can kill or interfere the growth of bacteria, and are used both in animals and humans for control or treat infections.^1,2^ In meat-producing animals, the most common problems due to antibiotics use are including bovine pneumonia, shipping fever and diarrhea,^3^ respiratory diseases, liver abscesses and improvement in growth.^4^ On the other hand, studies reported some safety adverse reactions for antibiotics including anaphylaxis, cardiotoxicity, nephrotoxicity, neurotoxicity and hepatotoxicity, and also documented number of hematological and gastrointestinal problems.^5^ Therefore, in relation to antimicrobial consumption in animal or human, some important items should be considered, including drug characterization (toxicity, pharmacodynamics, pharmacokinetics, cost and tissue distribution), the age and immune system of animal or human and appropriate drug dose which can be achieved by anti­microbial susceptibility test.^6^ According to studies, antimicrobials can be used as growth enhancer in low subtherapuetic doses, but these doses cannot destroy the bacteria and allow them to achieve more resistance to the drug.^7^ Concerns about misuse or overuse of antimicrobials as nontherapeutic and appearance of drug resistance have arisen when antimicrobial dose increased to 100 percent in aquaculture in the 1994–2004.^8,9^ Antimicrobial resistance pointed to the situation that a microorganism shows resistance to a drug that was effective for its killing or destroying previously.^10^ Todays, this issue have significant effect on mortality and morbidity of humans each year and has reported that antibiotic-resistant bacteria caused death of 700,000 people globally and has predicted that this rate tend to enhance approximately to 10 million by 2050.^11^ Adaptation of bacteria to various environmental stresses such as antibiotics, approve that they are quite adaptive organisms.^10^

There are two types of mechanisms for creation and spreading the resistant bacteria population: vertical gene transfer and horizontal gene transfer. The former, which is also called *intrinsic* resistance, occurs in evolutionary phase and genetic errors accumulate in the plasmid or chromosome of bacterial cells. However in the horizontal gene transfer or *acquired* resistance, the exchanges are within and between bacterial species in which the organisms gain new genes on their mobile genetic elements including plasmids, insertion sequences, phage-related elements and integrons, transposons.^12^

Antibacterial resistance can be spread by food chain through direct or indirect exposure. Direct exposure occurs, following the contact of human with animal or its blood, saliva, milk, semen, feces and urine which is very simple and rapid way for spreading resistant bacteria. The indirect contact occurs, following by consumption of contaminated food products such as egg, meat and dairy products which is more complex and far-reaching pathway.^13-15^ The other particular transport routs are related to environment which can be the source of antibiotic-resistance genes.^16,17^ As a result, the bacteria as a reservoir of resistance genes in addition of their pathogenicity, can be a hidden hazard for public health. The appearance of antimicrobial resistance by the food chain is a cross-sectorial problem; the first, antibiotics are extensively used in veterinary, aquaculture and agriculture, the second, antibacterial-resistant bacteria and genes can simply spread at each step of the food chain, and the last can be related to infectious diseases in humans.^18^ On the other hand, antibacterial-resistance can have globally dissemination by food chain due to extension of population, international travels and trade in food products.^12^ In preparing food animals, vegetables and fishes, in different ecosystems with numerous bacteria, large types of antibiotics are used which can cause to appearance resistant bacteria.^19^

Todays, antibiotic-resistance, especially that which is transferred from food chain to human is a global concern, and a lot of researches have been conducted to find approaches for solving this critical problem. In the present study, we tried to express some approaches for preventing the appearance of drug-resistant bacteria.

## Drug resistant bacteria & food safety

Food safety is a scientific course which has been focused on prevention and controlling the food borne diseases in all processes of the food production process including transport, storage, handling, preparation and in ensuring health and safety of foods for human consumption.^20^

Resistant food-borne diseases are one of the most important public health problems associated with the risk of emergence of antibacterial resistance in the food production chain. Literatures have indicated that increase in antibiotic resistance bacteria has been caused to an augment in food borne diseases.^21^ Besides, it should be noted that two–third of severity illnesses were related to Gram negative resistant bacteria which the importance and treatment ways of different resistant bacteria are dissimilar.^22^ Todays, different types of resistant bacteria have been identified in food products and humans, however, some basic and simple food safety measures such as appropriate hand-washing, convenient vegetable-washing, effective cooking temperatures and food storage situation can efficiently reduce and control the spread of antibacterial resistance foodborne pathogens.^20^ According to Mensah et al,^23^ antibiotic residues in food products can have different adverse effects for public health including allergic reactions, hepatotoxicity, mutagenicity, carcinogenicity, toxic effects, nephropathy and antibacterial resistance.

In overall, the results of spreading antibiotic-resistant bacteria and infectious diseases could be summarized as: (1) delay or unsuccessfulness in treatment, (2) limiting in selection of antimicrobials, (3) surviving of resistant strains in treatment of other bacterial illnesses, (4) coexistence and increased pathogenicity of resistance genes in result of selection.^24-26^ According to literatures, there are various food borne pathogens which indicates resistance to different drugs and antibiotics. Some important resistant food borne pathogens including:

Thermotolerant *Campylobacter*: its related disease has short duration with low mortality rate and public health problem. Studies found that some* Campylobacter* spp. were resistant to macrolides, quinolones, chloramphenicol, ampicillin, tetracy­cline, lincosamides, aminoglycosides and other tylosin, β-lactams and cotrimoxazole.^27,28^

*Salmonella:* one of the food borne pathogens which is very high risk factor for human health with remarkable worldwide distribution.* Salmonella* spp. have indicated multidrug resistance toward tetracyclines, kanamycin, sulfonamides, chloramphenicol, streptomycin, cephalosporins and penicillins.^29^

*Staphylococcus aureus* spp. are common pathogens for animals and human which reported as resistant-pathogens to penicillins as early as 1948.^30^ These resistant pathogens are important in dairy product.

*Enterococci* spp. are common bacteria in the intestinal tract of birds and mammals and known as indicators for enteric contamination of foods. These pathogens can endure unfavorable conditions such as low or high pH, temperature and saline waters^31,32^ which reveals that resistant-*Enterococci* can be an important factor of community-acquired infections.^33^ Furthermore, resistant *Enterococci* can transfer resistance gene to human-adapted strains and have adverse effect, indirectly.^32^

*Yersinia*: This genus is composed of various species, including *Y. pestis*, *Y. enterocolitica* and *Y. pseudotuberculosis*, which are pathogenic strains.^34^ *Y. enterocolitica* may lead to septicemia, septic arthritis, pneumonia, cellulitis, meningitis, empyema, osteomyelitis and panophthalmitis. According to literatures, strains of biovar 1B (serovar O:8) indicated resistance to carbenicillin, cephalothin and ticarcillin,^35^ whereas, biovar 3 (0:1,2,3 and O:5,27) strains were resistant to cefoxitin and amoxicillin/clavulanic acid.^36^

Recent publications where the food borne disease, resistant pathogen and their resistance mechanisms have been reported are shown in Table 1.

**Table 1:** A few food borne pathogens, related disease, antimicrobials and resistance mechanism

**Pathogen**	**Related disease symptoms**	**Antimicrobial group**	**Resistance mechanism**	**Ref.**
*Mycobacterium tuberculosis*	Tuberculosis	Fluoroquinolones	Modifying enzymes, target mimicry	^37^
*Streptococcus pneumonia*	Pneumococcal meningitis	Penicillin	Genetic alteration of penicillin-binding protein	^38^
*Vibrio cholerae*	Severe watery diarrhea	Sulfonamides	Chromosomal alterations in encoding dihydropteroate synthase	^39^
		Tetracycline	Preventing binding of the antibiotic	^40^
*Shigella dysenteriae*	Severe diarrhea	Chloramphenicol, tetracycline	Decreased permeability, efflux	^41^
*Salmonella thyphi*	Typhoid	Chloramphenicol	Alteration in target site, production of chloramphenicol acetyltransferase, active efflux	^42^
*Campylobacter jejuni*	Gastrointestinal illnesses	Tetracycline	Target protection, Change in ribosomal conformation and preventing binding of the antibiotic	^43^
*Candida krusei, C. albicans, C. glabrata*	Yeast infections, oral Thrush	Azoles	Alteration in ergosterol sites, incorporation of different sterols in plasma membrane, reduction in membrane permeability, effect on efflux pumps	^44^
*Streptococcus spp*	Sore throat, scarlet fever	Tetracycline	Target protection, Change in ribosomal conformation and preventing binding of the antibiotic	^43^
*Enterobacter, Serratia, Pseudomonas, Citrobacter*	‏-	B-lactam antibiotics	Decrease permeability cell membrane	^45^
		Quinolone	Alteration in DNA gyrase, antibiotic efflux systems	^46^
		Aminoglycoside	Cell-wall impermeability, enzymatic modification.	^47^
*P. aeruginosa*	Blood infection, necrotizing enterocolitis	Carbapenem	Mutational loss of porin channel, acquired zinc b-lactamase	^48^
*Enterococci*	Soft tissue infections	Vancomycin	Bypass of antibiotic target	^49^
*Staphylococcus aureus*	Vomiting, diarrhea, dehydration	Methicillin,	Related to *mecA* gene, decrease affinity of all b-lactams	^50^
		Streptogramin antibiotics, macrolide, lincosamide	Inactivating enzymes, modification of target sites, active efflux	^51^
		Quinolone	Active efflux, changing in DNA topoisomerases	^52^
*Neisseria Meningitidis*	Pyogenic, meningitis and septicemia	Chloramphenicol	Production of chloramphenicol acetyltransferase	^53^
		Rifampin	Alteration in RNA polymerase, membrane permeability	^54^
		Sulfonamides	Chromosomal alterations in encoding dihydropteroate synthase	^55^
		Penicillin	Alterations in penicillin-binding proteins	^53^

## Preventive approaches

### Farming practices

**Figure 1:**
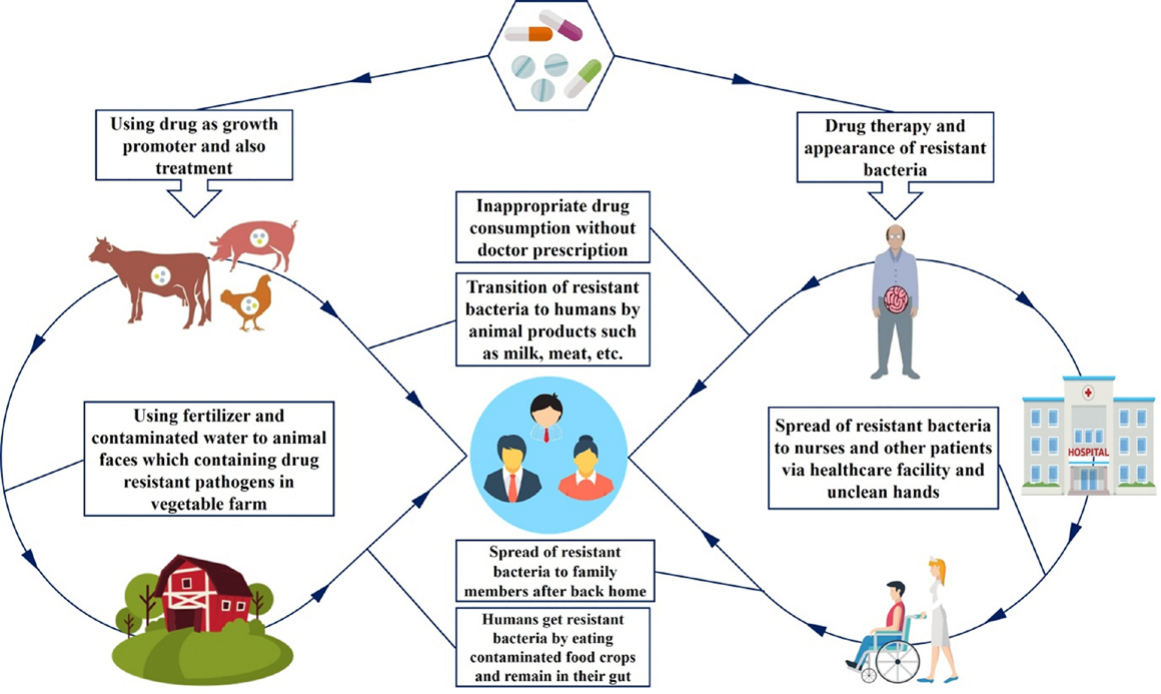
The cycle of emergence and remaining of resistant bacteria (adopted by https://apua.org/science-of-resistance).

Biosecurity measures in agriculture and food products have significant role in reduction of antimicrobial resistant bacteria and its transmission from farm to fork. According to the FAO and the WHO, 2011, biosecurity is defined as the measures for reduce or eliminate the threat of the emergence or spreading of diseases at region or country-levels.^56,57^ These measures which called as expressions such as hazard analysis and critical control points, good agricultural practices, good veterinary practices, good hygiene practices focus on health and management/assessment of microbiological risk.^56,57^ Therefore, biosecurity can play economical role in public health control plans, especially in agricultural production, because good and correct practice, well-controlled farming and using low chemical materials , leads to healthy animals and reduction in requirement of antibiotic treatments. On the other hand in the situation of appearance of resistant bacteria, effective practices can prevent its dissemination and ensure the food safety and food health.^58-60^ According to Österberg et al,^61^ organic plants indicated a significantly lower amount of resistant bacteria of *E. coli* rather than conventional ones. Figure 1 indicates the ways of appearance of drug- resistant bacteria in society.

Beside, rapid diagnostics of resistant bacteria and their genes will aid to agricultural practitioners to early detect and separate the infectious plants and animals and prevent of its quick spread.^1^ Almost, few of rapid tools such as PCR and microarray can identify the pattern of resistance and aid to define appropriate treatment methods.^62^ In applying these diagnostic tools, it should be considered that these methods must be rapid, fundamental, repeatable and easy to use.^12^

Todays, a lot of innovative and modern methods have been suggested for rapid and reliable detection of resistance bacteria in food process chain including, Fourier Transform Infrared Spectroscopy, ATR-FTIR,^63^ Nano scale materials (such as gold nano-wire), magnetic nanoparticles which are based on bacterial metabolic activity and also antibiotic susceptibility in blood or milk.^64^

## Alternatives for antibiotics for food animals

### Natural antibiotics

As previous expressed, todays injudicious use of drugs and especially antibiotics is the most important reason in the appearance of drug resistance thus a lot of studies have investigated using the natural and effective antimicrobial agents as alternative and complementary therapy method.^65^ Essential oils are biological and active substances which are produced by plants and can have antibacterial, antifungal, sedative, antioxidant, digestive, anticancer, anti-inflammatory and antiviral activities.^66-68^ The efficiency of these substances depends on their genotypes, chemical composition, agronomic and environmental conditions.^69^

Recent studies have revealed that essential oils can be used as preservative in foods and even can have prevention role in developing multidrug-resistant bacteria.^65,66,70^ According to literatures, some essential oils can have synergistic inhibitory effect in combination with conventional drugs which can lead to reducing effective dose of drugs and therefore, lessen their adverse effect.^71^ For example, study of Fadli et al,^72^ indicated that combination of conventional antibiotic and essential oil of Moroccan endemic thyme could have synergistic effect in antimicrobial activity and resulting in reduction of required effective dose, toxic side effects and also cost concerning of drug resistant bacteria. On the other hand, combination of essential oils with standard antibiotics can lead to different inhibition mechanisms in resistant bacteria and this can be a new choice to suppress the microbial resistance.^73^ Duarte et al,^71^ has studied on antimicrobial effect of classical antibiotics and coriander essential oil against A. baumannii and observed that the essential oil can improve antimicrobial effect of ciprofloxacin, gentamicin and tetracycline.

Essential oils are composed of many different terpenic compounds which lead to have various health effects. These biological substances have been used in different dosage forms including capsules, creams, aerosols, ointments, syrups, sprays and suppositories.^69^

However, essential oils have more advantages as antibiotic alternative, but their utility is limited by very low solubility in water and their sensitivity to oxygen, heat and moisture. Although many researches try to solve these problems. For example, nano-sized formulation or nano-encapsulation of these substances can be viable solutions against these problems.^74,75^

Table 2 reports recent publications where the effect of using classical antibiotics with essential oils are shown.

**Table 2:** Fractional inhibitory concentration (FIC) indices of classical and natural antimicrobial pairs against antibiotic resistant bacteria

**Combination**	**FIC**	**FICI**	**Target bacteria**	**Effect**	**Ref**
Meropenem–peppermint	-	0.26	*E. coli* J53 pMG321	Synergistic	^76^
Meropenem (µg/mL)	0.13				
Peppermint (%, v/v)	0.13				
Cefoperazone-coriander	-	0.750	*Acinetobacter baumannii* 1025	No interaction	^71^
Cefoperazone (µg/mL)	0.500				
Coriander oil(%, v/v)	0.250				
Erythromycin-eugenol	‏-	< 0.50	*Proteus vulgaris* NCIM 2813	Synergistic	^77^
Ampicillin-eugenol	‏-	1	*Staphylococcus aureus* blaZ	No interaction	^78^
Gentamicin-tea tree	‏-	0.5	*Acinetobacter baumannii* ATCC 19606	Synergistic	^79^
Meropenem-cinnamon	‏-	1.5	*Acinetobacter baumannii*	No interaction	^80^
Chloramphenicol-coriander	-	0.312	*Acinetobacter baumannii* 1025	Synergistic	^71^
Chloramphenicol (µg/mL)	0.062				
Coriander(%, v/v)	0.250				
Tetracycline- Lemon thyme	-	0.95–1.08	*Pseudomonas aeruginosa* ATCC 27853	No interaction	^81^
Meropenem–tea tree	-	1	*E. coli* J53 pMG321	No interaction	^76^
Meropenem (µg/mL)	0.50				
Tea tree (%, v/v)	0.50				
Tetracycline-cinnamaldehyde	‏-	0.37	*E. coli* N00666	Synergistic	^78^
Chloramphenicol-coriander	-	0.047	*Acinetobacter baumannii* 1041	Synergistic	^71^
Chloramphenicol (µg/ml)	0.016				
Coriander(%, v/v)	0.031				
Gentamicin-rosewood	‏-	0.11	*Acinetobacter baumannii* ATCC 19606	Synergistic	^79^
Piperacillin- Coriander Piperacillin (µg/mL) Coriander(%, v/v)	-	0.625	*Acinetobacter baumannii* 1041	No interaction	^71^
	0.125				
	0.500				
Penicillin-carvacrol	‏-	0.32	Salmonella Typhimurium SGI 1	Synergistic	^78^
Ampicillin-eugenol	‏-	<0.50	*Enterobacter aerogenes* NCIM 5139	Synergistic	^77^
Penicillin-Allyl isothiocyanate	‏-	0.66	*E. coli* SGI1	No interaction	^78^
Meropenem-lemon	‏-	2	*Acinetobacter baumannii*	No interaction	^80^
Tetracycline-coriander	-	0.185	*Acinetobacter baumannii* 1041	Synergistic	^71^
Tetracycline (µg/mL)	0.125	-			
Coriander oil (%, v/v)	0.062	-			
Cefixime-thyme	‏-	1.25	*Escherichia coli* ATCC 25922	No interaction	^82^
Cefixime-thyme	‏-	1	*Klebsiella pneumoniae*	No interaction	^72^
Ampicillin-thymol	‏-	0.12	*Escherichia coli* N00 666	Synergistic	^78^
Amoxicillin-sandarac	‏-	1	*Escherichia coli* ATCC 10536	No interaction	^83^
Chloramphenicol-geraniol	‏-	0.32–0.87	*Klebsiella pneumonia* ATCC 700603	Synergistic	^84^
Ceftazidime–cinnamon bark	-	2	*E. coli* J53 pMG309.	No interaction	^76^
Ceftazidime (µg/mL)	1	-			
Cinnamon bark (%, v/v)	1	-			

FIC of oil = MIC of oil in mixing by antibiotic/MIC of oil alone. FIC of antibiotic = MIC of antibiotic in mixing by oil/MIC of antibiotic alone. FIC index = FIC of oil + FIC of antibiotic.

## Nano-antibiotics

Classical antibiotic therapy lead to antibiotic resistance and this can prevent by using nano-antibiotics.^85^ “Nano-antibiotics” defined as all nano sized materials which have additional antimicrobial activity and can augment the overall efficacy and safety in consumption.^64^ Recent researches have been observed that using nano encapsulation system can improve efficacy of antibiotics.^86,87^

On the other hand, nano-carriers indicated functional role which can improve drug absorption by enhancing solubility, preventing from drug degradation, controlling intracellular penetration and etc.^88^ Today, biological nano-carrier systems such as liposomes and chitosan based capsules have been used as drug carrier because of their advantages (including biodegradability, economically, biocompatibility, having no or minimal side effects).^89^ The other advantage of nano-antibiotics is their functional properties by several approaches such as (*i*) producing different antibiotics by the same nanoparticles, (*ii*) using different mechanisms to prevent bacteria growth, (*iii*) improving in drug efflux, (*iv*) releasing high amount of antibiotics at the infection site.^64,90^ Therefore, manufacture and development of nano-antibiotics, in addition of prevention of emerging resistant bacteria and reduction the side effects of drugs, can facilitate their storage for long time which can be more economic, although, this science needs investment for developing and becoming commercial technology.

## Synthetic biology

Synthetic biology is defined as new patch in using of genome synthesis technologies to generate novel living systems with functional application.^91^ This new approach is offered for the improvement and exploitation of natural products to suppress the emergence of antibiotic resistant pathogens. Novel products are small molecules with genetical codes that have chemical structures which can be used as new antimicrobials.^91^ This state-of-art technology can design more cost-effective antibiotics with novel and specific activity, which connect biological systems to engineering processes. With emergence of resistance mechanisms, the antibiotics which are created based on random mutations, facing to obsolesce and till now only a few drugs are new and producing by pharmaceutical sector. Synthetic biology is based on building and integrating gene molecules for reaching to desired results. These biological and functional units can be used for biomedical applications.^92,93^

Biological systems can generate new drugs relied on the intelligent approaches, such systems are composed by products of biosynthetic genes for new production pathways. This technology can define biological machineries for different bacteria to predict their ability in production of antibiotics and designing synthetic bacteria and also new antibiotics with beneficial functionality by engineered control systems.^94^

Furthermore, this engineering framework, has potential uses in countering bacterial infections, biofuel production, synthesis of natural products and industrial chemicals substitutes.^92,95,96^ According to reports of genome sequencing of various fungi and bacteria, different species of microorganisms can be used for production of antibiotic.^91^ Although, as any developing technology, synthetic biology has some problems which leads to limit its application in industry. Fortunately, in antibiotics discovery, these limitations are far less than other fields.^91^ In the application of this technology, it should be considered to microbe safety in production of new antibiotics, security of drug discovery in fermentation tanks and their isolation and to economic concerns of synthetic biology in biosynthetic production of antibiotics.

In biosynthetic gene clusters some antimicrobial compounds including nonribosomal peptides, alkaloids, polyketide antibiotics, bacteriocins, saccharides and terpenoids^97^ which are used for encoding, can control the pathogenic microorganisms.^98^ Todays, by advancing in synthetic biology, DNA sequencing and synthesis, it is possible to access more diversity of antimicrobials and study on these organisms without laboratory culture.^99^

Chu et al., by using nonribosomal peptide synthetases and advanced synthetic biology, could produce new antimicrobials against methicillin-resistant *S. aureus*, which called humimycins, and enhanced the effect of β-lactam antibiotics.^100^ In overall, synthetic biology have an important role in steps of design, production and modification of natural products.^101^ Improvement in gene expression and developing strong regulators and promoters can be useful to produce larger and advanced compounds in future.

## Lactic acid bacteria

Lactic acid bacteria can be the other alternative approach for traditional antibiotics. According to literatures the foods containing probiotics, prebiotics, synbiotics have significant role in human and animal health.^62^ Probiotics or in particular lactic acid bacteria, are a group of Gram-positive, non-spore forming and acid tolerant organisms which are used as reformer of texture and taste in fermented foods.^102,103^ These bacteria are characterized by reduction in redox potential and pH, producing lactic acids, diacyls, bacteriocins, hydrogen peroxide, etc., which can degrade mycotoxins, prevent the growth and activity of pathogenic and food spoilage bacteria.^104,105^ Furthermore, according to recent studies, kimchi (traditional fermented food) caused by Lactobacillus plantarum LBP-K10, indicated high antifungal activity and also antiviral activity.^106,107^ The other solutions for preventing or treatment of diseases is association with gut bacterial microecosystem which can improve by prebiotics, probiotics, and synbiotics foods.^108,109^ Probiotics can protect the gut microbial flora, improve immune system and prevent colonization of pathogens.^110^ Therefore, finding new technologies for identifying and characterizing new strains of these bacteria with more antimicrobial activities can have effective role in protection of public health.

### Bacteriocin

As previous, LAB and their bacteriocins have indicated antimicrobial activities against various pathogens and can be used as effective replacement for antibiotics and other chemicals in the food technology. Bacteriocins are a group of potent antimicrobial peptides which compete with related and mostly Gram-positive organisms to gain more nutrients.^111^ These primary metabolites are small and cationic molecules with 30–60 amino acids that are heat stable at 100°C for 10 minutes and are different in mode of action, genetic origin, molecular weight (MW) and biochemical properties.^112^

In concept of anti-bacterial resistant bacteria, some novel properties of LAB and bacteriocins including site-specific delivery of drugs and strategies of anti-quorum sensing can have fascinating roles which lead to increase their application in future. Nisin and pediocin, two bacteriocins isolated from fermented foods, have been approved as natural preservatives by the FDA.^112^ Some of these antibacterial substances can be effective on both Gram-negative and Gram-positive food borne bacterial pathogens which can be used as biopreservatives and be important in preying human pathogens. Action site of bacteriocins is the cytoplasmic membrane of bacteria.^104^ Their drawback is associated to the inhibition effect of probiotics against mainly more related organisms and even desirable bacteria as starter cultures. Furthermore, they often have no activity on gram-negative food spoilers and pathogens but according to Kwak et al,^106,113^ using chelating agents can increase susceptibility of gram-negative bacteria to bacteriocins. According to recent study, heterofermentative *Lactobacillus* spp. can reduce the harmful microorganisms in Dutch-type cheese production, control human pathogens and therefore can affect on safety, quality and shelf life of food products.^114^

In spite of the excellent properties of these metabolites, it has been shown that resistant gene could have been horizontal transferred to benefit bacteria (that produce bacteriocin) in uncontrolled in vitro and in vivo analyses.^112^ Therefore, more researches need to ensure the efficacy and safety of these bacteria and their bacteriocins for health claims and clinical application.

### Cyclopeptide

Antimicrobial peptides are a group of metabolites of *Lactobacillus spp.* which are considered as an approach for novel pharmaceutical applications. These bioactive agents are some small substances including cyclic dipeptides and 2,5-diketopiperazines.^115^ Borthwick suggested that their inhibitory effect can be related to the presence of double bonds in amino acid residues at the α, β-positions of cyclic dipeptides, and also the NH proton in pyrroline and diketopiperazine ring.^115^ Dipeptidyl cyclic rings have been introduced as signal molecules which could reduce virulence-factor and prevent microbial growth for few decades_._^116,117^ These peptides such as some diketopiperazines (2,3-, 2,5-, and 2,6-diketopiperazines and their derivatives) indicate inhibitory effect against fungi, Gram- negative and - positive bacteria.^118^ According to Kwak et al,^107^ cis-cyclo (L-Phe-L-Pro) and cis-cyclo (L-Leu-L-Pro) have inhibitory effect on influenza A virus and also cis-cyclo (L-Phe-L-Pro) and cis-cyclo (L-Val-L-Pro) are active against growth of *Ganoderma boninense* and *Candida albicans* in plant and human, respectively.^106^

Liu et al^119^ reported some of these peptides were isolated from kimchi (a Chinese fermented food) and had antifungal and antibacterial effects. Studies showed that antioxidant peptides have some functional roles and suggested a novel microbial diketopiperazine from the cyclo (His-Leu) which produced by *Bacillus subtilis.*^120,121^ Researchers reported that cyclo (His-Pro) plus high doses of zinc have anti-hyperglycemic effect and stimulating consumption of muscle glucose^122^ and reduce obese diabetic (ob/ob) which can have significant positive effect on human health.^123^ Furthermore, Cyclo (Phe-Phe) can inhibit the development of dementia and Alzheimer’s disease by preventing acetylcholinesterase and serotonin transporter.^124^ According to Lee *et al.,*^125^ Cyclo (Trp-Trp) from some strains of *Streptomyces* indicated significant inhibitory effect on multi drug resistance of *Acinetobacter baumannii* and the other fungal and bacterial strains. Lind et al,^126^ observed antifungal activity against *Rhodotorula mucilaginosa* and *Aspergillus fumigatus* by cyclo (Ile-Pro) and Cyclo (Phe-Pro).

Therefore, antimicrobial peptides can be an alternative for traditional antibiotics, also these substances indicate bioactive activities, such as antiviral, antifungal, antitumor, antiprion and glycosidase inhibitor activities.^127^ However, some of these components have shown toxic effects on mammalian cells.^128^

## Immunostimulant

Vaccination is an infection preventive strategy which is based on immunological memory and body immune response to the foreign agent.^129^ Vaccines are inactivated or attenuated pathogens which can have protection role in again exposure to the same pathogen in future.^130,131^ In modern medicine, antibiotics and vaccines are used as the two greatest measures for prevention and treatment. The vaccines can suppress the antibacterial resistant bacteria by reducing the pathogen population which cause to reduce the level of antibacterial use and appearance of antibacterial resistant bacteria.^132^ Vaccines have prevalence role on resistant bacteria by direct and indirect mechanisms. They reduce the use of antibiotics in individuals for the same pathogen and also help to prevent transmission of disease to others. Furthermore, vaccines can affect on non-bacterial pathogens which can associate to superinfects and need to medicine treatments.^133^ Unfortunately, there are no certain treatment for many infectious diseases for example malaria, HIV, tuberculosis and salmonella which can lead to global problem.^134^ Vaccination treatment of these diseases may need more cost but can be an economical solution in long term and can inhibit the drug resistance which in turn protect millions of lives. The use of vaccination as an immunization program is a preventing method in animals and can significantly increase the productivity. According to studies, using lower antibiotics with appropriated novel vaccines program will be able to lessen the worldwide spread of infective diseases.^130,133^

## Bacteriophage

Bacteriophages are specifically viruses which act against bacteria as an alternative for antibiotics with breaking cell wall for solving appearance of resistance in bacteria.^135^ Bacteriophages with DNA or RNA genomes can encode endolysin enzymes that lysis cell wall by cleavage peptidoglycan. Besides, Bacteriophages genome encode proteins that are called amurins which inhibit cell wall synthesis resulting in breakage cell wall.^136^

Efforts for using bacteriophage for treatment is related to before discovering discovery of antibiotics.^137^ Bacteriophages have two lysogenic and lytic life cycles which have potential role in treatment options.^135^ A characteristic of bacteriophages is their specificity that can only act against targeted bacteria without any adverse effect on normal flora which is very important for improvement of health. On the other hand, this specificity lead to some problems in immunity issue of phage therapy and besides need to high specific procedures.^138^ Furthermore, this specificity causes problem for infections which are generally colonized by different strains of bacteria.^139^ Although a few studies confirmed the safety of oral administration of phage,^140,141^ the important issue is correct phage translocation across the intestinal epithelium.^142^ Of course, studies indicated this translocation can be useful for the body by regulation of immune system to indigenous microbe antigens through the prevention of tumor necrosis factor, interleukin-2 and interferon gamma production.^142^ However, some other studies did not observe significant increase in levels of cytokine through phage treatment.^143^ Despite of limited data about phage therapy, literatures have reported that phage therapy in addition to reduction of the gut pathogenic flora, causes very lower perturbation in comparison to traditional antibiotics.^144,145^

Regional specificity lead to finding phages with the highest infectivity against the target pathogen. This can be more helpful when looking for phages for antibiotic resistant bacteria especially in hospital.^146^ Moreover, phages have some enzymes such as extracellular polymeric substances depolymerase which can degrade bacterial biofilms and extracellular polymeric substances but antibiotic cannot treat the infection of biofilm-based bacteria.^147^ Gabisoniya et al^148^ observed that phages inhibited additional biofilm formation and also destroyed existing biofilm of the *Pseudomonas aeruginosa.*

## Predatory bacteria

The last alternative solution against resistant bacteria infections is the use of some bacteria known as ‘’predatory bacteria’’. *Micavibrio aeruginosavorus*, *Bdellovibrio bacteriovorus* and associated organisms are gram-negative bacteria and belong to two subgroup of proteobacteria.^149,150^ These bacteria due to their proteases and DNases, demonstrated potential in predation of various pathogenic bac­teria and are not specific against gram-negative bacteria.^115,134,135^ The interesting factor of these predatory bacteria is that they can prey effectively even bacterial biofilms and multidrug-resistant pathogens including *E. coli*, *P. aeruginosa, A. baumannii*, *Pseudomonas putida* and *Klebsiella pneumonia*.^110,151,152^ Predatory bacteria could serve both as probiotic and antibiotic organisms.^10^ Studies reported that these bacteria are effective in treatment of ocular diseases caused by *Shigella flexneri* and *Moraxella bovis* in rabbits and cows, respectively.^153^ With the development of antibacterial resistant bacteria and inadequately treatment by conventional antibiotics, predatory bacteria as live antibacterials can have effective role in human health and treatment of diseases.^153,154^

In spite of useful properties of these bacteria, they have some limitations in their application. Predatory bacteria can have negative effect on the natural microbiota of the body.^155^ On the other hand, they may have incomplete predation on bacteria and remain a few number of bacteria.^151^ Furthermore, existence of Gram-positive bacteria can reduce their predation efficacy.^151^

## Conclusion

Drug resistant bacteria are a global concern phenomenon which can have adverse effects on public health and economy. The assurance of food safety from farm to fork is significantly affected by inappropriate use of drugs and especially antibiotics as treatment agents and growth promoters in food animals. Judicious use of antibiotics has been led to the emergence of anti-bacterial resistant pathogens which can contaminate the food products, reach human body and cause different problems to health. For solving this issue, many researchers have investigated on alternatives for antibiotics in farm practice and also human treatment. Simple biosecurity measures in agriculture and food production process are really beneficial practices which have significant role in food safety and transmission of drug resistant bacteria to humans. Furthermore, combination therapy, combining essential oils and traditional antibiotics, as a new method in pharmacology can be an effective and economic approach against antibiotic resistance. However, these methods need more consideration, encouragement and investment. The discovery and development of novel drugs with new technologies as alternatives to classical antibiotics is a vital issue in global health.

## Ethical Issues

Not applicable.

## Conflict of Interest

Authors declare no conflict of interest in this study.

## Acknowledgments

This study is related to the project NO. 1397/ 71592 stimulated to Student Research Committee, Shahid Beheshti University of Medical Sciences, Tehran, Iran. We also appreciate the “Student Research Committee” and “Research & Technology Chancellor” in Shahid Beheshti University of Medical Sciences for their financial support of this study.
